# Towards harmonisation of case definitions for eight work-related musculoskeletal disorders - an international multi-disciplinary Delphi study

**DOI:** 10.1186/s12891-021-04871-9

**Published:** 2021-12-04

**Authors:** Sietske J. Tamminga, P. Paul F. M. Kuijer, Kathryn Badarin, Jose Hernán Alfonso, Joana Amaro, Stefania Curti, Irina Guseva Canu, Stefano Mattioli, Ingrid S. Mehlum, David Rempel, Yves Roquelaure, Steven Visser, Henk F. van der Molen

**Affiliations:** 1 Department of Public and Occupational Health, Amsterdam UMC, University of Amsterdam, Coronel Institute of Occupational Health, Netherlands Center for Occupational Diseases, Amsterdam Public Health Research Institute, Meibergdreef 9, Amsterdam, The Netherlands; 2 Unit of Occupational Medicine, Institute of Environmental Medicine, Karolinska Institutet, Stockholm, Sweden; 3 Department of Occupational Medicine and Epidemiology, National Institute of Occupational Health, Oslo, Norway; 4 EPIUnit - Institute of Public Health, University of Porto, Porto, Portugal; 5 Department of Medical Sciences, University of Ferrara, Ferrara, Italy; 6 Department of Occupational and Environmental Health, Center of Primary Care and Public Health (Unisanté), University of Lausanne, Lausanne, Switzerland; 7 Institute of Health and Society, University of Oslo, Oslo, Norway; 8 Division of Occupational and Environmental Medicine, University of California, San Francisco, USA; 9 Univ Angers, CHU Angers, Univ Rennes, Inserm, EHESP, Irset (Institut de recherche en santé, environnement et travail) - UMR_S 1085, F-49000 Angers, France

**Keywords:** Low back pain, Lumbosacral radicular syndrome, Subacromial pain syndrome, Carpal tunnel syndrome, Lateral elbow tendinopathy, Medial elbow tendinopathy, Epicondylitis, Tennis elbow, Knee osteoarthritis, Hip osteoarthritis, Occupational disease

## Abstract

**Background:**

International consensus is needed on case definitions of work-related musculoskeletal disorders and diseases (MSDs) for use in epidemiological research. We aim to: 1) study what information is needed for the case definition of work-related low back pain (LBP), lumbosacral radicular syndrome (LRS), subacromial pain syndrome (SAPS), carpal tunnel syndrome (CTS), lateral and medial elbow tendinopathy, and knee and hip osteoarthritis, and to 2) seek consensus among occupational health professionals/researchers regarding the case definitions of these work-related MSDs.

**Methods:**

A two-round Delphi study was conducted with occupational health professionals/researchers from 24 countries. Definition of work-related MSDs were composed of a case definition with work exposures. Round 1 included 32 case definitions and round 2, 60 case definitions. After two rounds, consensus required 75% of the panellists to rate a case definition including work exposures ≥7 points on a 9-point rating scale (completely disagree/completely agree).

**Results:**

Fifty-eight panellists completed both rounds (response rate 90%). Forty-five (70%) panellists thought that for LBP a case definition can be based on symptoms only. Consensus was only reached for work-related medial elbow tendinopathy, while the lowest agreement was found for knee osteoarthritis. Where consensus was not reached, this was – except for LBP - related to physical examination and imaging rather than disagreement on key symptoms.

**Conclusion:**

Consensus on case definitions was reached only for work-related medial elbow tendinopathy. Epidemiological research would benefit from harmonized case definitions for all MSDs including imaging and physical examination for LRS, SAPS, CTS, lateral elbow tendinopathy and hip and knee osteoarthritis.

**Supplementary Information:**

The online version contains supplementary material available at 10.1186/s12891-021-04871-9.

## Background

Prevention of work-related musculoskeletal disorders and diseases (MSDs) is supported by evidence of a medical diagnosis of a disorder/disease, valid assessment of work-related exposure and the knowledge about the association between work-related exposure and the disease/disorder [1, 2]. To give a practical example: a lateral elbow tendinopathy due to playing tennis in leisure time cannot clinically be distinguished from a lateral elbow tendinopathy due to high hand grip forces at work [1]. Therefore, it is pivotal to understand the nature and extent of the occupational exposure to be able to manage and prevent work-related lateral elbow tendinopathy [3, 4].

A case definition is typically a set of symptoms, signs and diagnostic tests that is used to establish a diagnosis of a disease/disorder [5]. Some MSDs can be considered a disease - usually defined as “a particular distinctive process in the body with a specific cause and characteristic symptoms” - while other MSDs can be considered a disorder, usually defined as “irregularity, disturbance or interruption of normal functions” [1]. A case definition can be used in clinical care, in research, or in surveillance. Whether a case definition needs high sensitivity, high specificity or both may depend on its purpose, and it may furthermore, depend on the setting and resources [6, 7].

Variation in MSD case definitions used in occupational cohort studies can lead to large variations in the occurrence estimates for work-related MSDs [1, 8–10] and may hamper our knowledge of associations between work-related exposures and MSDs [9, 11].

In a prior scoping review, we assessed the degree of consensus on case definitions for eight predefined MSDs, namely: non-specific low back pain, lumbosacral radicular syndrome, subacromial pain syndrome, carpal tunnel syndrome, lateral and medial elbow tendinopathy, and knee and hip osteoarthritis [6]. These MSDs were selected based on their high frequency in the working population. In this study, we identified only one case definition for lateral and medial elbow tendinopathy and concluded that less variation in case definitions was found for non-specific low back pain compared to the other included MSDs [6]. The variation in the other MSDs was related to which physical examination(s) and imaging was needed and heterogeneity in signs and symptoms [6]. In addition, it was reported that work-related criteria were included in two studies only [6]. International harmonisation of case definitions of work-related MSDs for use in epidemiological occupational research will improve the ability to compare findings and pool data between studies [12]. This, in turn, will improve estimates of rates of MSDs and work-related risk factors, both of which are important for the prevention and management of work-related MSDs.

Therefore, the objectives of this study were: i) to study what minimum information is needed for a case definition of eight work-related MSDs that can be used in epidemiological research, ii) to seek consensus among occupational health professionals/researchers regarding the case definitions of eight work-related MSDs.

## Methods

### Design

A Delphi technique was used to seek consensus regarding the case definition of eight work-related MSDs, which was justified because of the large variation in MSD case definitions found in our scoping review [6]. The Delphi technique aimed to reduce the amount of variation in these case definitions and highlight the remaining differences. We used the criteria proposed by Jünger et al. [13] to report the present Delphi study. The case definition of a work-related MSD includes the medical symptoms and signs, plus exposure to occupational risk factors that are known to be associated with the incidence of these MSDs. These diseases/disorders were: (chronic) non-specific low back pain, lumbosacral radicular syndrome, subacromial pain syndrome, carpal tunnel syndrome, lateral and medial elbow tendinopathy, and knee and hip osteoarthritis. We defined a priori that consensus would be reached when at least 75% of the panellists rated a case definition or work exposure ≥7 on a 9-point rating scale (1 = completely disagree to 9 = completely agree). This cut-off value was based on a review of Delphi studies in which a cut-off for consensus was often set at 75% [13]. The Delphi technique consisted of two rounds by the same panellists. In the case consensus was not reached after two rounds, recommendations for further research were formulated. We restricted the procedure to two rounds as more rounds would most likely lead to selective attrition as it has been suggested that bias may be introduced by lower response rates [14]. LimeSurvey (www.limesurvey.com) was used to circulate the Delphi questionnaires and to collect data. Both Delphi questionnaires were pilot tested by the research team as to formulation and content. Data were analysed using SPSS software (IBM SPSS Statistics version 24). The protocol was not published or deposited in an open access repository before starting the study, but the protocol was discussed within the Network on the Coordination and Harmonisation of European Occupational Cohorts (OMEGA-NET) [12] working group and was not altered during the study. This study did not fall under the scope of the Medical Research Involving Human Subjects Act [15] in the Netherlands, and did not need ethical approval [15, 16]. The first author [ST] was the main coordinator of the Delphi study and sent the invitations to the panellists to fill in the questionnaires in both Delphi rounds. She was not involved in the OMEGA-NET nor an expert on work-related MSDs thereby limiting the chance to directly or indirectly influence the panellists’ judgements.

### International panellists

The inclusion criteria for participating in the Delphi study were: i) being an occupational health professional and/or researcher in occupational health, ii) having expertise on work-related MSDs, and iii) being able to read and write in English. The inclusion criteria were not assessed but were self-reported. Panellists were recruited by sending an email to OMEGA-NET [12]. To be able to participate in this network you have to be a national scientific and appointed expert on this topic. In this email, each OMEAGA-NET member was asked to participate if they fulfilled the eligibility criteria, and/or to nominate at least one eligible occupational health professional and/or researcher in occupational health with expertise on work-related MSDs from their country of residence.

### Procedure

**Fig. 1:**
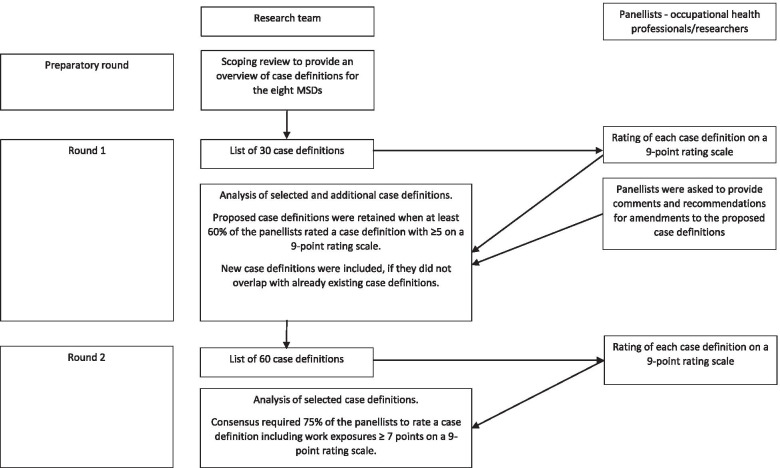
Flow chart

The procedure is shown in Fig. 1. The panellists’ demographics (i.e. country, age, sex, occupation) were recorded in Delphi round 1. Reminders were sent to panellists who did not respond. One email reminder was sent in Delphi round 1 after two weeks and two email reminders in Delphi round 2, after 1.5 and 3.5 weeks, respectively. At the end of each Delphi round, plain collective results were provided to the panellists.

#### Preparatory round

A set of proposed case definitions for each MSD were extracted from the aforementioned scoping review when the study reported diagnostic criteria that were based on: 1) expert consensus, 2) a guideline based on a systematic literature review, or 3) a synthesis of the literature [6]. The aim of this scoping review was to provide an overview of case definitions of diagnostic criteria for eight MSDs for use in occupational healthcare, surveillance or research [6].

#### Round 1

First, for each MSD, panellists were asked whether they were of the opinion that a case definition for epidemiological research can be based on symptoms only (yes/no).

Second, Panellists were asked to rate each case definition on a 9-point rating scale (1 = completely disagree 9 = completely agree) (Additional file 1: Table 1). For some MSDs this entailed several proposed case definitions. For each of the eight MSDs, the panellists were also asked to provide comments and recommendations for amendments to the proposed case definitions (Additional file 1: Table 1).

Finally, the proposed work exposures for each of the eight MSDs were extracted from the aforementioned scoping review [6] and from the Dutch registration guidelines for the reporting of occupational MSDs, which are based on systematic literature reviews [17, 18]. Panellists were asked to rate each proposed work exposure for each of the eight MSDs on the same 9-point rating scale. For each of the eight MSDs, they were also asked to provide comments and recommendations for amendments on the proposed work exposure (Additional file 1: Table 2).

#### Round 2

First, for each MSD, panellists were asked to give their opinion on the minimum information that should be included in a case definition for use in epidemiological research where performing a physical examination or imaging are not possible. The options were: self-reported symptoms (yes/no), self-report that a diagnosis was made by a physician (yes/no), self-reported limitation of daily activities (yes/no), self-reported limitation of work activities (yes/no) (multiple answers possible), or none of the above is sufficient (yes/no).

Second, as in round 1, panellists were asked to rate each proposed case definition on a 9-point rating scale (1 = completely disagree 9 = completely agree). Proposed case definitions were retained from Delphi round 1 when at least 60% of the panellists rated a case definition with ≥5 on a 9-point rating scale. These criteria were pre-determined. In addition, the percentage of the panellists who rated a case definition ≥5 on a 9-point scale during the first round was displayed alongside each case definition. In addition, new case definitions were included based on results from the open-ended questions in Delphi round 1, if they did not overlap with already existing case definitions.

Finally, panellists were asked to rate each work exposure on the same 9-point rating scale. The same procedure as described above for case definitions was followed here.

## Results

### Panellists

The invitation to the OMEGA-NET members to participate and/or nominate at least one eligible occupational health professional and/or researcher from their country of residence yielded 79 persons to invite. Out of these invited persons, 64 completed Delphi round 1 (participation rate 81%) and 58 of these 64 panellists completed Delphi round 2 (response rate 90%). The panellists were from 24 countries, with a mean age of 47 years, most of them were female (*N* = 37; 58%) and most worked as occupational physicians (N = 37; 58%) (Table 1).

**Table 1:** Characteristics of the panellists (*N* = 64)

Socio-demographic characteristics			
Age in years *(mean ± SD)*			47.2 ± 10.3
Sex *(N(%) female)*			37 (58%)
Country of residency *(N(%))*		Italy	12 (19%)
		Turkey	6 (10%)
		Netherlands	5 (8%)
		USA	5 (8%)
		Macedonia	3 (5%)
		Portugal	3 (5%)
		Romania	3 (5%)
		Latvia	3 (5%)
		Switzerland	3 (5%)
		France	2 (3%)
		Hungary	2 (3%)
		Belgium	2 (3%)
		Moldova	2 (3%)
		Luxembourg	2 (3%)
		Norway	2 (3%)
		Other or missing	9 (12%)
**Work-related characteristics**			
Current occupation *(N(%)*)^a^	Occupational health professional	Occupational physician	37 (58%)
		Ergonomist	10 (16%)
		Rehabilitation physician	5 (8%)
		Physical therapist	4 (6%)
		Occupational therapist	2 (3%)
		Other	13 (20%)
	Researcher	Epidemiologist	12 (19%)
		Other	37 (58%)
Combination of occupations *(N(%)*)	Both researcher and occupational health professional		26 (40%)
	Occupational health professional		26 (40%)
	Researcher		12 (20%)
Number of years of experience in current occupation *(mean ± SD)*^b^			18.1 ± 10.4
OMEGA-NET member *(N(%) yes)*			32 (50%)

^a^numbers do not add up to 100% as one panellist may have several occupations

^b^Formulated as ‘how many years of experience do you have in your current occupation?’

### Objective 1 - minimum information needed for a case definition for use in epidemiological research

In Delphi round 1, 70% (*N* = 45) of panellists agreed that for the purposes of epidemiological research, a case definition of low back pain could be based solely on symptoms. The proportion of agreement was similar for chronic low back pain (69%, *N* = 44) (Table 2).

**Table 2:** Minimum information that should be included in a case definition for use in epidemiological research

Disorder/disease	Delphi round 1 (***N*** = 64). A case definition for epidemiological research can be based on symptoms only (yes(%))	Delphi round 2 (***N*** = 58). The minimum information that should be included in a case definition for use in epidemiological research when it is not possible to perform physical examination or imaging (yes (%)).				
		Self-reported symptoms N(%)	Self-report that a diagnosis was made by a physician N(%)	Self-reported limitation of daily activities N(%)	Self-reported limitation of work activities N(%)	None of the above is sufficient N(%)
**Chronic low back pain**	44 (69%)	39 (67%)	24 (41%)	33 (57%)	32 (55%)	4 (7%)
**Low back pain**	45 (70%)	44 (76%)	21 (36%)	31 (53%)	28 (48%)	1 (2%)
**Lumbosacral radicular syndrome**	23 (36%)	37 (64%)	34 (59%)	30 (52%)	26 (45%)	4 (7%)
**Carpal tunnel syndrome**	23 (36%)	36 (62%)	32 (55%)	22 (38%)	25 (43%)	5 (9%)
**Subacromial pain syndrome**	22 (34%)	NR	NR	NR	NR	NR
**Later or medial elbow tendinopathy**	27 (42%)	34 (59%)	31 (53%)	27 (47%)	26 (45%)	8 (14%)
**Knee osteoarthritis**	12 (19%)	33 (57%)	31 (53%)	31 (53%)	24 (41%)	9 (16%)
**Hip osteoarthritis**	14 (22%)	31 (53%)	31 (53%)	31 (53%)	22 (38%)	9 (16%)

NR not recorded in Delphi round 2 due to a technical mistake

In Delphi round 2, most panellists considered that, the minimum information that should be included in a case definition for use in epidemiological research, is self-reported symptoms (Table 2). For lateral and medial elbow tendinopathy and knee and hip osteoarthritis, approximately half of the panellists had the opinion that self-reported symptoms should be included. For these disorders, similar percentages indicated that a case definition should at a minimum include “a self-report that the diagnosis was made by a physician” and “self-reported limitation pof daily activities” (Table 2). Note that for subacromial pain syndrome the answers to ‘what the minimum information that should be included in a case definition for use in epidemiological research’ was not recorded in Delphi round 2 due to a technical mistake.

### Objective 2 and 3– level of agreement regarding the case definition and work exposure of eight MSDs for epidemiological research

#### Non-specific low back pain

A previously proposed case definition of non-specific low back pain *“acute or recurrent low back pain”* was not retained after the first Delphi round (Additional file 1: Table 1). Based on the open-ended question in Delphi round 1, three new case definitions were added (Additional file 1: Table 1). The case definition *“ months of pain, muscle tension or stiffness localized below the costal margin and above the inferior gluteal folds, without leg pain* “reached the highest level of agreement (*N* = 38; 68% rated ≥7 on a 9-point rating scale) (Table 3).

**Table 3:** Case definition with the highest agreement and work exposures for which consensus was reached - results of Delphi round 1 (*N* = 63) and Delphi round 2 (*N* = 56)

Disease/disorder		Delphi Round 1		Delphi Round 2	
		Number of panellists who rated a case definition ≥5 on a 9-point rating scale (N(%)).	Median (range)	Number of panellists who rated a case definition ≥7 on a 9-point rating scale (N(%)).	Median (range)
**Non-specific low back pain**					
Case definition	< 3 months of pain, muscle tension or stiffness localized below the costal margin and above the inferior gluteal folds, without leg pain.	53 (84%)	7 (1–9)	38 (68%)	7 (2–9)
Work exposure	A. Manual handling of loads.	61 (97%)	8 (4–9)	**52 (93%)**	8 (4–9)
	B. Whole-body vibration.	61 (97%)	7 (4–9)	**48 (86%)**	8 (4–9)
	C. Frequently bending and twisting of the trunk.	61 (97%)	8 (4–9)	**52 (93%)**	8 (5–9)
	D. Manual lifting.	57 (91%)	8 (3–9)	**52 (93%)**	8 (4–9)
**Lumbosacral radicular syndrome**					
Case definition	A pain with/without functional limitation, lasting less than 4 weeks (1 month), in the posterior region included between the inferior limit of the costal arch and the inferior buttock fold, with posterior irradiation below the knee or anterior to the thigh. Leg pain can be present even without lumbar pain.	57 (91%)	7 (2–9)	39 (70%)	7 (3–9)
Work exposure	A. Manual handling of heavy loads.	61 (97%)	7 (4–9)	**52 (93%)**	8 (4–9)
	B. Bending or twisting of the trunk.	58 (92%)	7 (2–9)	**48 (86%)**	8 (5–9)
	C. Whole-body vibration.	NA	NA	**42 (75%)**	7 (3–9)
	D. Manual lifting.	NA	NA	**46 (82%)**	8 (4–9)
**Subacromial pain syndrome**					
Case definition	All signs/symptoms below: 1. Intermittent shoulder pain without paresthesia. 2. Pain worsened by active elevation movement of the upper arm as in scratching of the upper back. 3. Symptoms present now or on at least 4 days during the last 7 days.	60 (95%)	7 (4–9)	38 (68%)	7 (3–9)
Work exposure	A. Arm elevation (hand at or above shoulder height).	62 (98%)	8 (4–9)	**45 (80%)**	8 (5–9)
	B. Combination of: 1. Arm elevation (hand at or above shoulder height). 2. Repetitive work with hand and/or arm. 3. Daily work with vibrating hand tools. 4. High force of upper extremity.	NA	NA	**51 (91%)**	8 (4–9)
**Carpal tunnel syndrome**					
Case definition	All signs/symptoms below: Intermittent paresthesia or pain in at least 2 of digits I, II or III. Either may be present at night as well (allowing pain in the palm, wrist, or radiation proximal to the wrist). Symptoms present now or on at least 4 days during the last 7 days.	55 (87%)	7 (3–9)	41 (73%)	7 (4–9)
Work exposure	A. Repetition (frequency of exertion and duty cycle of exertion) of wrist/hand/fingers.	61 (97%)	8 (4–9)	**53 (95%)**	8 (5–9)
	B. Force (peak effort exerted by the hand).	61 (97%)	7 (3–9)	**51 (91%)**	8 (4–9)
	C. Combined exposures (repetition + force).	60 (95%)	8 (1–9)	**54 (97%)**	9 (6–9)
	D. Vibration of the hand/arm.	60 (95%)	8 (4–9)	**49 (88%)**	8 (3–9)
**Lateral elbow tendinopathy**					
Case definition	All signs/symptoms below: 1. Intermittent, activity dependent pain directly located around the lateral epicondyle. 2. Symptoms present now or on at least 4 days during the last 7 days. 3. Local pain on resisted wrist extension (lateral). 4. Pain exacerbated when holding a coffee cup.	NA	NA	40 (71%)	7 (2–9)
Work exposure	Combination of turn and screw	61 (97%)	7 (3–9)	**48 (86%)**	8 (5–9)
	Repetitive bending and twisting of the elbow	60 (95%)	7 (3–9)	**49 (88%)**	8 (3–9)
	High physical exertion combined with elbow movements	57 (91%)	7 (3–9)	**45 (80%)**	8 (4–9)
	High physical exertion of the wrist combined with elbow movements.	NA	NA	**42 (75%)**	7 (2–9)
**Medial elbow tendinopathy**					
Case definition	All signs/symptoms below: 1. Intermittent, activity dependent pain directly located around the medial epicondyle. 2. Symptoms present now or on at least 4 days during the last 7 days 3. Local pain on resisted wrist flexion (medial). 4. Tenderness on direct pressure.	NA	NA	**42 (75%)**	7.5 (2–9)
Work exposure	A. High hand grip forces	59 (94%)	7 (4–9)	**45 (80%)**	8 (4–9)
	B. Repetitive movements	61 (97%)	7 (4–9)	**42 (75%)**	7 (1–9)
	A. A combination of: 1.Handling loads 2.High hand grip forces 3.Repetitive movements 4. Working with vibrating hand tools	NA	NA	**42 (75%)**	8 (3–9)
**Knee osteoarthritis**					
Case definition	Pain in the knee and at least three of the six symptoms assessed by physical examination /signs/personal factors: 1. Age ≥ 50 years 2. Stiffness < 30 min. 3. Crepitus. 4. Pain at palpation knee bone. 5. Bone deformation (X-ray). 6. No palpable warmth.	NA	NA	35 (63%)	7 (1–9)
Work exposure	A. Kneeling and/or squatting	62 (98%)	8 (3–9)	**52 (93%)**	8 (4–9)
	B. Jumping	60 (95%)	7 (3–9)	**48 (86%)**	8 (4–9)
	C. Climbing the stairs/ladder	58 (92%)	7 (1–9)	**48 (86%)**	8 (5–9)
	D. Heavy Lifting	NA	NA	**44 (79%)**	8 (4–9)
**Hip osteoarthritis**					
Case definition	Combination of: 1. Pain distal thigh or even medial knee region. 2. Limited range of motion. 3. Disability. 4. Morning stiffness < 1 h. 5. Degeneration (X-ray)	NA	NA	36 (64%)	7 (2–9)
Work exposure	A. Heavy lifting	58 (92%)	7 (2–9)	**46 (82%)**	8 (1–9)

*NA*: not applicable as these case definitions or work exposure were suggested in Delphi round 1 to be added to Delphi round 2. In bold consensus reached (consensus was a priori defined when ≥75% of the panellists rated a case definition with ≥7 on a 9-point rating scale)

All four working exposures were retained after Delphi round 1 and reached the pre-defined consensus level (Table 3). Ten new work exposures were added based on the open-ended question in Delphi round 1, but none reached the pre-defined consensus level in Delphi round 2 (Additional file 1: Table 2).

#### Lumbosacral radicular syndrome

A previously proposed case definition of lumbosacral radicular syndrome, namely *“Monoradicular leg pain”* was not retained after the first Delphi round (Additional file 1: Table  1). Based on the open-ended question in Delphi round 1, nine new case definitions were added that specified symptom characteristics (Additional file 1: Table 1). The case definition “*A pain with/without functional limitation, lasting less than 4 weeks (1 month), in the posterior region including between the inferior limit of the costal arch and the inferior buttock fold, with posterior irradiation below the knee or anterior to the thigh. Leg pain can be present even without lumbar pain “*reached the highest level of agreement (*N* = 39; 70% rated ≥7 on a 9-point rating scale) (Table 3).

Two work exposures were retained after Delphi round 1 and reached the pre-defined consensus level (Table 3). Two new work exposures were added based on the open-ended question in Delphi round 1, and these two reached the pre-defined consensus level in Delphi round 2 as well (Table 3, Additional file 1: Table 2).

#### Subacromial pain syndrome

A previously proposed case definition of subacromial pain syndrome *“Shoulder pain and/or weakness”* was not retained after the first Delphi round (Additional file 1: Table  1). Based on the open-ended question in Delphi round 1, four new case definitions were added, that provide greater specificity on symptoms (Additional file 1: Table  1). The case definition “*All of the following signs/symptoms: 1. Intermittent shoulder pain without paraesthesia, 2. Pain worsened by active elevation movement of the upper arm as in scratching of the upper back, 3. Symptoms present now or on at least 4 days during the last 7 days”* reached the highest level of agreement (*N* = 38; 68% rated ≥7 on a 9-point rating scale) (Table 3).

All work exposures were retained after Delphi round 1; however, only “Arm elevation (hand at or above shoulder height)” reached the pre-defined consensus level (Table 3). Four new work exposures were added based on the open-ended question in Delphi round 1, of which one reached the pre-defined consensus level in Delphi round 2 (Table 3, Additional file 1: Table 2).

#### Carpal tunnel syndrome

All previously proposed definitions were retained after the first Delphi round (Additional file 1: Table  1). Based on the open-ended question in Delphi round 1, four new case definitions were added mainly specifying symptoms and the use of imaging (Additional file 1: Table 1). The case definition: “*All of the following symptoms/signs: Intermittent paraesthesia or pain in at least 2 of digits I, II or III, either may be present at night as well (allowing pain in the palm, wrist, or radiation proximal to the wrist), symptoms present now or on at least 4 days during the last 7 days”* reached the highest level of agreement (*N* = 41; 73% rated ≥7 on a 9-point rating scale) (Table 3).

All work exposures were retained after Delphi round 1 and four reached the pre-defined consensus level (Table 3). Two work exposures, were added, based on the open-ended question in Delphi round 1, but they did not reach the pre-defined consensus level in Delphi round 2 (Additional file 1: Table 2).

#### Lateral elbow tendinopathy

The single proposed case definition was retained after the first Delphi round (Additional file 1: Table 1). Based on the open-ended question in Delphi round 1, two new case definitions were added that included specific symptoms and the use of imaging (Additional file 1: Table 1). The case definition “*All of the following signs/symptoms: 1. Intermittent, activity-dependent pain directly located around the lateral epicondyle, 2. Symptoms present now or on at least 4 days during the last 7 days, 3. Local pain on resisted wrist extension (lateral), 4. Pain exacerbated when holding a coffee cup”* reached the highest level of agreement (*N* = 40; 71% rated ≥7 on a 9-point rating scale) (Table 3).

All work exposures were retained after Delphi round 1 of which three reached the pre-defined consensus level (Table 3). Eight work exposures were added based on the open-ended question in Delphi round 1, of which one reached the pre-defined consensus level in Delphi round 2 (Table 3, Additional file 1: Table 2).

#### Medial elbow tendinopathy

The single proposed case definition was retained after the first Delphi round (Additional file 1: Table 1). Based on the open-ended question in Delphi round 1, two new case definitions were added that included functional limitations (Additional file 1: Table 1). Only for the case definition “All of the following signs/symptoms: *1. Intermittent, activity-dependent pain directly located around the medial epicondyle, 2. Symptoms present now or on at least 4 days during the last 7 days*, *3. Local pain on resisted wrist flexion (medial), 4. Tenderness on direct pressure”* consensus was reached of all case definitions (*N* = 42; 75% rated ≥7 on a 9-point rating scale) (Table 3).

All work exposures were retained after Delphi round 1, of which two reached the pre-defined consensus level (Table 3). Four working were added based on the open-ended question in Delphi round 1, of which one reached the pre-defined consensus level in Delphi round 2 (Table 3, Additional file 1: Table 2).

#### Knee osteoarthritis

All previously proposed case definitions were retained after the first Delphi round (Additional file 1: Table 1). Based on the open-ended question in Delphi round 1, four new case definitions were added that mainly included the use of imaging (Additional file 1: Table 1). The case definition “*Pain in the knee and at least three of the six symptoms assessed by physical examination/signs/personal factors: 1. Age ≥ 50 years; 2. Stiffness < 30 minutes; 3. Crepitus; 4. Pain at palpation knee bone; 5. Bone deformation (X-ray); 6. No palpable warmth”* reached the highest level of agreement (*N* = 35; 63% rated ≥7 on a 9-point rating scale) (Table 3).

All work exposures were retained after Delphi round 1, of which three reached the pre-defined consensus level (Table 3). Four work exposures were added based on the open-ended question in Delphi round 1, of which one reached the pre-defined consensus level in Delphi round 2 (Table 3, Additional file 1: Table 2).

#### Hip osteoarthritis

Two previously proposed case definitions of hip osteoarthritis, namely “hip pain” and “(no abnormal) hip pain”, were not retained after the first Delphi round (Additional file 1: Table 1). Based on the open-ended question in Delphi round 1, five new case definitions were added that mainly included the use of physical examination and imaging (Additional file 1: Table 1). The case definition: “*A combination of: 1. Pain in the distal thigh or even medial knee region; 2. Limited range of motion; 3. Disability; 4. Morning stiffness < 1 hour; 5. Degeneration (X-ray)”* reached the highest level of agreement (*N* = 36; 64% rated ≥7 on a 9-point rating scale) (Table 3).

All work exposures were retained after Delphi round 1; however, only “heavy lifting” reached the pre-defined consensus level (Table 3). Six work exposures were added based on the open-ended question in Delphi round 1, but none of them reach the pre-defined consensus level in Delphi round 2 (Additional file 1: Table 2).

## Discussion

Consensus on a case definition was only reached for work-related medial elbow tendinopathy. Where consensus was not reached, this was – except for low back pain - related to the need for additional clinical assessment and imaging rather than disagreement on the key symptoms.

In contrast, to the scoping review mentioned in the introduction section [6], consensus on a case definition was only reached for work-related medial elbow tendinopathy. This was unexpected as in the scoping review we also identified only one case definition for lateral elbow tendinopathy and concluded that less variation in case definitions was found for non-specific low back pain [6]. This lack of consensus may be rooted in the method utilised. In this Delphi study our sample of panellists consisted of various health and research disciplines from different countries. This might have introduced bias and heterogeneity as their professional training and beliefs about the medical diagnoses and work-related causes might differ. Nonetheless, this Delphi methodology is intended as a first step to find consensus and describe remaining perceived differences in work-related MSD case definitions among occupational health professionals. Clearly, further research is needed to identify consensus criteria for most of the MSDs for which consensus could not be reached. Furthermore, only two Delphi rounds were employed in order to prevent panellist drop-out. Another Delphi round might have achieved greater consensus, especially as so many new case definitions were proposed in Delphi round 1 and as for some MSDs (i.e. knee and hip osteoarthritis) there was less consensus after round 2 compared to round 1 (Additional file 1: Table 1). Finally, the lack of consensus for the seven MSDs may also be a result of greater variation in the existing case definitions for these MSDs compared to the case definitions for medial and lateral elbow tendinopathy. In Delphi round 1, we had one case definition for the elbow tendinopathies compared to three to seven case definitions for the other MSDs. In Delphi round 2, there were three case definitions for the elbow tendinopathies compared to six to thirteen for the other MSDs. Finally, another possible explanation may be that the case definition for medial elbow tendinopathy might need less sensitivity and specificity since there are fewer differential diagnoses to consider for elbow pain.

For further research, to reach consensus on the other seven MSDs, it may be useful to subdivide the consensus process into first reaching consensus on physical examination and imaging, followed by reaching consensus on symptoms and clinical signs and finally on combining both in a case definition. The consensus process on the signs and symptoms may be preceded by a semantic analysis to get a narrower proposal of signs and symptoms before the start of the Delphi process; this was successfully applied to reach consensus on the case definition of occupational burn out [19].

Except for low back pain, panellists were of the opinion that a case definition for MSDs for use in occupational epidemiological research, should not be based on self-reported symptoms only. This is in contrast with current research practice. For example, a sub inventory seeking information on MSD case definitions used in 21 European cohort studies [12] found that in 6 out of 11 cohorts that provided information the MSD case definition was based on both self-assessment and clinical assessment (i.e. physical examinations, imaging, and / or diagnostic techniques) [20–25]. These clinical assessments included physical examinations in three cohorts [22, 23, 25] and data from medical records in another three cohorts [20, 21, 24]. In the Octopus cohort study on carpal tunnel syndrome the case definition was also based on electromyography exams [25, 26]. As it might be challenging to include clinical assessment in cohort studies [7], this suggests, that, for future epidemiological research, additional strategies such as imaging reports provided by participants may be considered in case imaging reports could not be directly produced by the study researchers [27].

Most work exposures that were added to Delphi round 2 based on the open-ended questions of Delphi round 1 did not reach consensus in round 2. This might be an indication of the quality of the systematic reviews and may display the most commonly used concepts. Since we found consensus on work exposures, a next step would be to reach consensus on threshold values per work exposure in terms of level, duration and/or frequency in order to distinguish between high and low health risks [1]. These threshold values should be validated for each work-related MSD, in a high-quality cohort study using valid and reliable exposure assessments like quantitative measurements of the exposure or video-based observations [2].

## Conclusions

For use in epidemiological research, consensus on a case definition was reached only for work-related medial elbow tendinopathy. Future epidemiological research would benefit from harmonized case definitions for the other 7 MSDs including signs from imaging and physical examination for lumbosacral radicular syndrome, subacromial pain syndrome, carpal tunnel syndrome, lateral elbow tendinopathy and hip and knee osteoarthritis.

## Additional file

**Additional file 1: Table 1.** Agreement with each case definition for use in epidemiological research - results of Delphi round 1 (*N*=63) and Delphi roud 2 (*N*=56). **Table 2.** Results of Delphi round 1 (*N*=63) and round 2 (*N*=56) on work exposures.

## Data Availability

The datasets used and/or analysed during the current study are available from the corresponding author on reasonable request.

## Abbreviations

MSDs: Musculoskeletal disorders and diseases
OMEGA-NET: Network on the Coordination and Harmonisation of European Occupational Cohorts
