# Supplementary material for: Towards harmonisation of case definitions for eight work-related musculoskeletal disorders - an international multi-disciplinary Delphi study
Source: BMC Musculoskelet Disord. 2021 Dec 4;22:1018. doi: 10.1186/s12891-021-04871-9 (PMC8645098; doi:10.1186/s12891-021-04871-9)
Supplement: Supplementary file 1 — Additional file 1: Table 1. Agreement with each case definition for use in epidemiological research - results of Delphi round 1 (N=63) and Delphi roud 2 (N=56). Table 2. Results of Delphi round 1 (N=63) and round 2 (N=56) on work exposures. [file 12891_2021_4871_MOESM1_ESM.docx]

**Table 1 - Agreement with each case definition for use in epidemiological research - results of Delphi round 1 (N=63) and Delphi roud 2 (N=56).**

**IN THE CASE ≥60% OF THE PARTICIPANTS RATED A CASE DEFINITION WITH ≥5, CASE DEFITINION WAS RETAINED.**

**IN THE CASE ≥75% OF THE PARTICIPANTS RATED A CASE DEFINITION WITH ≥7, consensus WAS reached.**

**Please rate your agreement with each non-specific low back pain case definition for use in cohort studies on a 9-point rating scale:**

| **Non-specific low back pain** | | **Delphi Round 1**  Number of participants who rated a case definition ≥5 on a 9-point rating scale (N(%)). | **Delphi round 1**  Median (range) | **Delphi Round 2**  Number of participants who rated a case definition **≥5** on a 9-point rating scale (N(%)). | **Delphi Round 2**  Number of participants who rated a case definition **≥7** on a 9-point rating scale (N(%)). | **Delphi round 2**  Median (range) |
| --- | --- | --- | --- | --- | --- | --- |
|  | Pain in the low back. | 38 (60%) | 5 (1-9) | 33 (59%) | 18 (32%) | 5 (1-9) |
|  | < 12 weeks of pain, muscle tension or stiffness in lower back without leg pain. | 53 (84%) | 7 (1-9) | 47 (84%) | 31 (55%) | 7 (1-9) |
|  | < 3 months of pain, muscle tension or stiffness localized below the costal margin and above the inferior gluteal folds, without leg pain. | 53 (84%) | 7 (1-9) | 51 (91%) | 38 (68%) | 7 (2-9) |
|  | A pain with/without functional limitation, lasting less than 4 weeks (1 month), in the posterior region included between the inferior limit of the costal arch and the inferior buttock fold, possibly with posterior irradiation to the thigh, but not below the knee. | 48 (76%) | 6 (2-9) | 50 (90%) | 29 (52%) | 7 (4-9) |
|  | Pain in low back. Pain bad enough to limit usual activities or change your daily routine for more than one day. | 48 (76%) | 6 (1-9) | 45 (80%) | 23 (41%) | 6 (1-9) |
|  | Acute or recurrent low back pain. | 33 (52%) | 5 (1-9) | NA | NA | NA |
|  | Back pain without leg pain in the forefront - resulting in limit usual activities or change your daily routine. | NA | NA | NA | 20 (36%) | 6 (1-9) |
|  | Intense pain in low back for more than 4 days in a row. | NA | NA | NA | 16 (29%) | 5 (1-9) |
|  | Pain in low back, muscle tension, loss of mobility and interference with usual daily activities. | NA | NA | NA | 29 (52%) | 7 (2-9) |

**Please rate your agreement with each lumbosacral radicular syndrome case definition for use in cohort studies on a 9-point rating scale:**

| **Lumbosacral radicular syndrome** | | **Delphi Round 1**  Number of participants who rated a case definition ≥5 on a 9-point rating scale (N(%)). | **Delphi round 1**  Median (range) | **Delphi Round 2**  Number of participants who rated a case definition **≥5** on a 9-point rating scale (N(%)). | **Delphi Round 2**  Number of participants who rated a case definition ≥7 on a 9-point rating scale (N(%)). | **Delphi round 2**  Median (range) |
| --- | --- | --- | --- | --- | --- | --- |
|  | Pain, muscle tension, or stiffness localized below the costal margin and above the inferior gluteal folds with leg pain. | 47(75%) | 6 (1-9) | 43 (77%) | 24 (43%) | 6 (1-9) |
|  | A pain with/without functional limitation, lasting less than 4 weeks (1 month), in the posterior region included between the inferior limit of the costal arch and the inferior buttock fold, with posterior irradiation below the knee or anterior to the thigh. Leg pain can be present even without lumbar pain. | 57 (91%) | 7 (2-9) | 48 (86%) | 39 (70%) | 7 (3-9) |
|  | Radicular pain in 1 lower limb. | 39 (62%) | 5 (1-9) | 38 (68%) | 13 (23%) | 5 (1-9) |
|  | Radiating pain, neuropathy, muscle weakness, incontinence, sensitivity. | 38 (60%) | 5 (1-9) | 35 (63%) | 17 (30%) | 5 (1-9) |
|  | Monoradicular leg pain. | 35 (56%) | 5 (1-9) | NA | NA | NA |
|  | Radicular pain in 1 lower limb. Pain bad enough to limit usual activities or change your daily routine for more than one day. | NA | NA | NA | 23 (41%) | 6 (1-9) |
|  | Radicular pain in 1 or both lower limbs. | NA | NA | NA | 11 (20%) | 5 (1-9) |
|  | Radiating pain, neuropathy, muscle weakness, urinary incontinence, sensitivity. | NA | NA | NA | 14 (25%) | 5 (1-9) |
|  | Radicular pain in 1 or both lower limb(s) either with hypo or hyper sensitivity of the leg. | NA | NA | NA | 16 (29%) | 5 (1-9) |
|  | A history of dermatomal leg pain, leg pain worse than back pain, worsening of leg pain during coughing, sneezing or straining, and straight leg raise test. Radiculopathy is characterised by the presence of weakness, loss of sensation, or loss of reflexes associated with a particular nerve root, or a combination of these, and can coexist with radicular pain. | NA | NA | NA | 33 (59%) | 7 (1-9) |
|  | A pain with/without functional limitation, lasting less than 4 weeks (1 month), in the posterior region included between the inferior limit of the costal arch and the inferior buttock fold, with posterior irradiation below the knee or anterior to the thigh. Leg pain associated with lumbar pain. | NA | NA | NA | 31 (55%) | 7 (3-9) |
|  | A pain with/without functional limitation, in the posterior region included between the inferior limit of the costal arch and the inferior buttock fold, with posterior irradiation below the knee or anterior to the thigh. Leg pain can be present even without lumbar pain and with or without sensory disturbances or weakness in the leg. | NA | NA | NA | 29 (52%) | 7 (2-9) |
|  | Radicular or monoradicular pain (electric type of pain) with incontinence. | NA | NA | NA | 12 (21%) | 4 (1-9) |
|  | Combination of lumbar pain and leg pain with symptoms such as numbness and hyposensitivity. | NA | NA | NA | 19 (34%) | 5 (1-9) |

**Please rate your agreement with each subacromial pain syndrome case definition for use in cohort studies on a 9-point rating scale:**

| **Subacromial pain syndrome** | | **Delphi Round 1**  Number of participants who rated a case definition ≥5 on a 9-point rating scale (N(%)). | **Delphi round 1**  Median (range) | **Delphi Round 2**  Number of participants who rated a case definition **≥5** on a 9-point rating scale (N(%)). | **Delphi Round 2**  Number of participants who rated a case definition ≥7 on a 9-point rating scale (N(%)). | **Delphi round 2**  Median (range) |
| --- | --- | --- | --- | --- | --- | --- |
|  | All signs/symptoms below:  1. Intermittent shoulder pain without paresthesia.  2. Pain worsened by active elevation movement of the upper arm as in scratching of the upper back.  3. Symptoms present now or on at least 4 days during the last 7 days. | 60 (95%) | 7 (4-9) | 54 (96%) | 38 (68%) | 7 (3-9) |
|  | Shoulder pain and/or weakness. | 29 (46%) | 4 (1-9) | NA | NA | NA |
|  | Shoulder pain, localized around the acromion, often worsening during or subsequent to lifting of the arm. | 54 (86%) | 6 (1-9) | 49 (88%) | 25 (45%) | 6 (2-9) |
|  | Combination of:  1. Shoulder pain (incl. night pain, pain at rest)  2. Stiffness  3. Loose or unstable shoulder  4. Weakness  5. Painful clicking, grinding, or clunking  6. Unusual sensations such as catching, locking, or grinding | 53 (84%) | 6 (1-9) | 48 (86%) | 23 (41%) | 6 (2-9) |
|  | Combination of:  1. Shoulder pain (incl. night pain, pain at rest)  2. Stiffness  3. Loose or unstable shoulder (as assessed by physical examination)  4. Weakness  5. Painful clicking, grinding, or clunking  6. Unusual sensations such as catching, locking, or grinding | NA | NA | NA | 26 (46%) | 6 (2-9) |
|  | Pain worsened by active elevation movement of the upper arm as in scratching of the upper back and/or from postural stress when lying in bed. | NA | NA | NA | 22 (39%) | 6 (1-9) |
|  | Shoulder pain, localized around the acromion, often worsening during or subsequent to lifting of the arm asensation of worsening with constant shoulder abduction | NA | NA | NA | 24 (43%) | 6 (1-9) |
|  | All signs/symptoms below:  1. Intermittent shoulder pain without paresthesia (incl. night pain, pain at rest).  2. Pain worsened by active elevation movement of the upper arm as in scratching of the upper back.  Stiffness 3. Loose or unstable shoulder 4. Weakness 5. Painful clicking, grinding, or clunking  6. Unusual sensations such as catching, locking, or grinding 7. Symptoms present now or on at least 4 days during the last 7 days. | NA | NA | NA | 29 (52%) | 7 (2-9) |

**Please rate your agreement with each carpal tunnel syndrome case definition for use in cohort studies on a 9-point rating scale:**

| **Carpal tunnel syndrome** | | **Delphi Round 1**  Number of participants who rated a case definition ≥5 on a 9-point rating scale (N(%)). | **Delphi round 1**  Median (range) | **Delphi Round 2**  Number of participants who rated a case definition **≥5** on a 9-point rating scale (N(%)). | **Delphi Round 2**  Number of participants who rated a case definition ≥7 on a 9-point rating scale (N(%)). | **Delphi round 2**  Median (range) |
| --- | --- | --- | --- | --- | --- | --- |
|  | All signs/symptoms below:  Intermittent paresthesia or (night)pain in at least 2 of digits I, II or III.  Either may be present at night as well (allowing pain in the palm, wrist, or radiation proximal to the wrist).  Symptoms present now or on at least 4 days during the last 7 days. | 55 (87%) | 7 (3-9) | 52 (93%) | 41 (73%) | 7 (4-9) |
|  | All signs/symptoms below:  1. Numbness and tingling in the median nerve distribution.  2. Numbness at night.  3. Weakness/atrophy of the thenar musculature. | 56 (89%) | 6 (1-9) | 49 (88%) | 33 (59%) | 7 (3-9) |
|  | All signs/symptoms below:  1. Brachialgia paraesthetica at night (Sleeping feeling in the hand).  2. "Electric shocks" by gripping movements or a permanent persistent sensation "the fingers tingle constantly". | 42 (67%) | 5 (1-9) | 41 (73%) | 18 (32%) | 5 (2-9) |
|  | All signs/symptoms below:  Numbness along the distribution of the median nerve increasing at night when sleeping.  Improvement by shaking the hands exacerbation when driving or holding a telephone. | 46 (73%) | 5 (2-9) | 44 (79%) | 23 (41%) | 6 (2-9) |
|  | All signs/symptoms below:  1. (at night, painful) Tingling, and/or numbness of the thumb, index finger,  middle finger, radial side of the ring finger and adjacent region of the palm.  2. Fluttering with hands can provide relief (‘flick-sign’)). | 46 (73%) | 6 (1-9) | 46 (82%) | 22 (39%) | 6 (1-9) |
|  | Numbness, tingling, and pain in the hands. | 46 (73%) | 4 (1-9) | 31 (55%) | 9 (16%) | 5 (1-9) |
|  | All signs/symptoms below:  1. Paraesthesia, pain, and numbness in the hands.  2. Pain in hand, wrist and forearm.  3. Atypical localization of tingling sensations. | 46 (73%) | 4 (1-9) | 35 (63%) | 15 (27%) | 5 (1-9) |
|  | All signs/symptoms below:  1. Intermittent paresthesia or (night)pain in at least 2 of digits I, II or III.  2. Either may be present at night as well (allowing pain in the palm, wrist, or radiation proximal to the wrist).  3. (at night, painful) Tingling, and/or numbness of the thumb, index finger, middle finger, radial side of the ring finger and adjacent region of the palm.  4. Fluttering with hands can provide relief (‘flick-sign’).  5. Symptoms present now or on at least 4 days during the last 7 days. | NA | NA | NA | 39 (70%) | 8 (2-9) |
|  | All signs/symptoms below:  1. Numbness along the distribution of the median nerve increasing at night when sleeping.  2. Improvement by shaking the hands exacerbation when driving or holding a telephone.  3. Functional limitation | NA | NA | NA | 27 (48%) | 6 (1-9) |
|  | All signs/symptoms below:  1. (at night, painful) Tingling, and/or numbness of the thumb, index finger, middle finger, radial side of the ring finger and adjacent region of the palm.  2. Fluttering with hands can provide relief (‘flick-sign’).  3. Muscle atrophy of the thenar musculature (EMG) | NA | NA | NA | 23 (41%) | 6 (2-9) |
|  | All signs/symptoms below:  1. Intermittent paresthesia or (night)pain in at least 2 of digits I, II or III.  2. Either may be present at night as well (allowing pain in the palm, wrist, or radiation proximal to the wrist).  3. Symptoms present now or on at least 4 days during the last 7 days  4. Axonopathy (Electroneuromyographic) | NA | NA | NA | 25 (45%) | 6 (3-9) |

**Please rate your agreement with each lateral elbow tendinopathy case definition for use in cohort studies on a 9-point rating scale:**

| **Lateral elbow tendinopathy** | | **Delphi Round 1**  Number of participants who rated a case definition ≥5 on a 9-point rating scale (N(%)). | **Delphi round 1**  Median (range) | **Delphi Round 2**  Number of participants who rated a case definition **≥5** on a 9-point rating scale (N(%)). | **Delphi Round 2**  Number of participants who rated a case definition ≥7 on a 9-point rating scale (N(%)). | **Delphi round 2**  Median (range) |
| --- | --- | --- | --- | --- | --- | --- |
|  | All signs/symptoms below:  1. Intermittent, activity dependent pain directly located around the lateral epicondyle.  2. Symptoms present now or on at least 4 days during the last 7 days  3. Local pain on resisted wrist extension (lateral). | 61 (97%) | 7 (2-9) | 53 (95%) | 39 (70%) | 7 (2-9) |
|  | 1. Intermittent, activity dependent pain directly located around the medial epicondyle. 2. Symptoms present now or on at least 4 days during the last 7 days 3. Local pain on resisted wrist flexion (lateral).  4. Constriction of the tendon (ultrasound). | NA | NA | NA | 27 (48%) | 6 (1-9) |
|  | All signs/symptoms below:  1. Intermittent, activity dependent pain directly located around the lateral epicondyle.  2. Symptoms present now or on at least 4 days during the last 7 days  3. Local pain on resisted wrist extension (lateral).  4. Pain exacerbated when holding a coffee cup. | NA | NA | NA | 40 (71%) | 7 (2-9) |

**Please rate your agreement with each medial elbow tendinopathy case definition for use in cohort studies on a 9-point rating scale:**

| **Medial elbow tendinopathy** | | **Delphi Round 1**  Number of participants who rated a case definition ≥5 on a 9-point rating scale (N(%)). | **Delphi round 1**  Median (range) | **Delphi Round 2**  Number of participants who rated a case definition **≥5** on a 9-point rating scale (N(%)). | **Delphi Round 2**  Number of participants who rated a case definition ≥7 on a 9-point rating scale (N(%)). | **Delphi round 2**  Median (range) |
| --- | --- | --- | --- | --- | --- | --- |
|  | All signs/symptoms below:  1. Intermittent, activity dependent pain directly located around the medial epicondyle.  2. Symptoms present now or on at least 4 days during the last 7 days  3. Local pain on resisted wrist flexion (medial). | 60 (95%) | 7 (2-9) | 52 (93%) | 37 (66%) | 7 (2-9) |
|  | All signs/symptoms below:  1. Intermittent, activity dependent pain directly located around the medial epicondyle.  2. Symptoms present now or on at least 4 days during the last 7 days  3. Local pain on resisted wrist flexion (medial).  4. Tenderness on direct pressure. | NA | NA | NA | 42 (75%) | 7.5 (2-9) |
|  | All signs/symptoms below:  1. Intermittent, activity dependent pain directly located around the lateral epicondyle.  2. Symptoms present now or on at least 4 days during the last 7 days  3. Local pain on resisted wrist extension (lateral).  4. Function limitations (e.g. holding fork and knife, tooth brushing). | NA | NA | NA | 35 (63%) | 7 (1-9) |

**Please rate your agreement with each knee osteoarthritis case definition for use in cohort studies on a 9-point rating scale:**

| **Knee osteoarthritis** | | **Delphi Round 1**  Number of participants who rated a case definition ≥5 on a 9-point rating scale (N(%)). | **Delphi round 1**  Median (range) | **Delphi Round 2**  Number of participants who rated a case definition **≥5** on a 9-point rating scale (N(%)). | **Delphi Round 2**  Number of participants who rated a case definition ≥7 on a 9-point rating scale (N(%)). | **Delphi round 2**  Median (range) |
| --- | --- | --- | --- | --- | --- | --- |
|  | Pain in the knee and at least three of the six symptoms/signs/personal factors: 1. Age ≥ 50 years  2. Stiffness < 30 minutes.  3. Crepitus.  4. Pain at palpation knee bone.  5. Bone deformation.  6. No palpable warmth. | 52 (83%) | 7 (1-9) | 43 (77%) | 27 (48%) | 6 (3-9) |
|  | A combination of:   1. Persistent knee pain. 2. Limited morning stiffness. 3. Reduced function. 4. Crepitus. | 57 (91%) | 6 (2-9) | 48 (86%) | 33 (59%) | 7 (3-9) |
|  | A combination of:  1. Knee joint pain.  2. Stiffness < 30 minutes.  3. Crepitus. | 47 (75%) | 6 (1-9) | 46 (82%) | 18 (32%) | 6 (3-9) |
|  | A. Two mandatory symptoms (knee pain in the absence of any recent trauma or injury and very short joint stiffness, lasting for less than 10 min, when starting movement) even in the absence of risk factors, OR  B. knee pain, and 1 or 2 risk factors (see below) OR  C. Three or more risk factors (see below) in the presence of at least one mandatory symptom, with symptoms lasting less than 6 months.  These criteria are applicable in the absence of active inflammatory arthritis, generalized pain, Kellgren- Lawrence grade >0, any recent knee trauma or injury, and age lower than 40 years.  Risk factors:  Overweight (body mass index >25)  Family history of OA  Previous knee injury  Malalignment  Lowerlimbs dissymmetry  OA in other sites  Metabolic syndrome  Not being ready to run or walk fast  after a period of inactivity | 54 (86%) | 7 (1-9) | 48 (86%) | 29 (52%) | 7 (2-9) |
|  | 1. Two out of the 4 KOOS subscales need to score positive (≤85%):  I. Knee pain  II. Symptoms, stiffness  III. Functioning, daily living  IV. Knee-related quality of life (QOL).  2. At least one present out of joint line tenderness or crepitus.  3. Degeneration. | 53 (84%) | 7 (1-9) | 49 (82%) | 25 (45%) | 6 (3-9) |
|  | Pain in the knee and at least three of the six symptoms assessed by physical examination /signs/personal factors: 1. Age ≥ 50 years  2. Stiffness < 30 minutes.  3. Crepitus.  4. Pain at palpation knee bone.  5. Bone deformation (X-ray).  6. No palpable warmth. | NA | NA | NA | 35 (63%) | 7 (1-9) |
|  | 1. Two out of the 4 KOOS subscales need to score positive (≤85%):  I. Knee pain  II. Symptoms, stiffness  III. Functioning, daily living  IV. Knee-related quality of life (QOL).  2. At least one present out of joint line tenderness or crepitus assessed by physical examination  3. Degeneration **(**X-ray**)**. | NA | NA | NA | 31 (55%) | 7 (1-9) |
|  | A. Two mandatory symptoms (knee pain in the absence of any recent trauma or injury and very short joint stiffness, lasting for less than 10 min, when starting movement) even in the absence of risk factors, OR  B. knee pain, and 1 or 2 risk factors (see below) OR  C. Three or more risk factors (see below) in the presence of at least one mandatory symptom, with symptoms lasting less than 6 months.  These criteria are applicable in the absence of active inflammatory arthritis, generalized pain, Kellgren- Lawrence grade >0 **(**X-ray**)**, any recent knee trauma or injury, and age lower than 40 years.  Risk factors:  Overweight (body mass index >25)  Family history of osteoarthritis  Previous knee injury  Malalignment  Lower limbs dissymmetry  Osteoarthritis in other sites  Metabolic syndrome  Not being ready to run or walk fast after a period of inactivity | NA | NA | NA | 29 (52%) | 7 (2-9) |
|  | A combination of:  1. Knee joint pain.  2. Stiffness < 30 minutes.  3. Crepitus.  4. feeling that knee give way when descending stairs. | NA | NA | NA | 25 (45%) | 6 (3-9) |

**Please rate your agreement with each hip osteoarthritis case definition for use in cohort studies on a 9-point rating scale:**

| **Hip osteoarthritis** | | **Delphi Round 1**  Number of participants who rated a case definition ≥5 on a 9-point rating scale (N(%)). | **Delphi round 1**  Median (range) | **Delphi Round 2**  Number of participants who rated a case definition **≥5** on a 9-point rating scale (N(%)). | **Delphi Round 2**  Number of participants who rated a case definition ≥7 on a 9-point rating scale (N(%)). | **Delphi round 2**  Median (range) |
| --- | --- | --- | --- | --- | --- | --- |
|  | A combination of:  1. Hip pain.  2. Limited range of motion.  3. Disability.  4. Morning stiffness < 1 hour. | 58 (92%) | 7 (1-9) | 48 (86%) | 35 (63%) | 7 (3-9) |
|  | Hip pain. | 28 (44%) | 4 (1-9) | NA | NA | NA |
|  | (no abnormal) Hip pain. | 21 (33%) | 3 (1-9) | NA | NA | NA |
|  | A combination of:  1. Pain distal thigh or even medial knee region and/or groin.  2. Limited range of motion.  3. Disability.  4. Morning stiffness < 1 hour. | NA | NA | NA | 31 (55%) | 7 (3-9) |
|  | A combination of:  1. Hip pain.  2. Limited range of motion.  3. Disability.  4. Morning stiffness < 1 hour.  5. Limited functional activities such as not able to reach to cut toenails | NA | NA | NA | 32 (57%) | 7 (3-9) |
|  | A combination of two or more of the following:  1. Hip pain  2. Limited range of motion  3. Reduction in physical capacity that limits standing walking or tolerance to high impact activities such as running and/or jumping  4. Stiffness in the morning or after a period of sitting | NA | NA | NA | 35 (63%) | 7 (3-9) |
|  | A combination of:  1. Mechanical Hip pain.  2. Limited range of motion.  3. Disability.  4. Morning stiffness < 1 hour. | NA | NA | NA | 23 (41%) | 6 (1-9) |
|  | A combination of:  1. Pain distal thigh or even medial knee region.  2. Limited range of motion.  3. Disability.  4. Morning stiffness < 1 hour.  5. Degeneration (X-ray) | NA | NA | NA | 36 (64%) | 7 (2-9) |

**Table 2 - Results of Delphi round 1 (N=63) and round 2 (N=56) on work exposures.**

**IN THE CASE ≥60% OF THE PARTICIPANTS RATED A WORK-RELATED CRITERION WITH ≥5, IT WAS RETAINED.**

**IN THE CASE ≥75% OF THE PARTICIPANTS RATED A CASE DEFINITION WITH ≥7, consensus WAS reached.**

| **Non-specific low back pain** | | **Delphi Round 1**  Number of participants who rated a symptom ≥5 on a 9-point rating scale (N(%)). | **Delphi round 1**  Median (range) | **Delphi Round 2**  Number of participants who rated a case definition ≥7 on a 9-point rating scale (N(%)). | **Delphi round 2**  Median (range) |
| --- | --- | --- | --- | --- | --- |
|  | Manual handling of loads. | 61 (97%) | 8 (4-9) | 52 (93%) | 8 (4-9) |
|  | Whole-body vibration. | 61 (97%) | 7 (4-9) | 48 (86%) | 8 (4-9) |
|  | Frequently bending and twisting of the trunk. | 61 (97%) | 8 (4-9) | 52 (93%) | 8 (5-9) |
|  | Manual lifting. | 57 (91%) | 8 (3-9) | 52 (93%) | 8 (4-9) |
|  | Bending of the trunk. | NA | NA | 37 (66%) | 7 (3-9) |
|  | Twisting of the trunk. | NA | NA | 35 (63%) | 7 (3-9) |
|  | Prolonged sitting. | NA | NA | 31 (55%) | 7 (1-9) |
|  | Overland truck driving. | NA | NA | 27 (48%) | 6 (1-9) |
|  | Riding lawn mowers | NA | NA | 21 (38%) | 6 (1-9) |
|  | Passive posture with repeated movements. | NA | NA | 24 (43%) | 6 (1-9) |
|  | Stress at the workplace. | NA | NA | 22 (40%) | 6 (1-9) |
|  | Little autonomy. | NA | NA | 19 (34%) | 5 (1-9) |
|  | Carrying. | NA | NA | 31 (55%) | 7 (3-9) |
|  | Pushing / pulling. | NA | NA | 25 (53%) | 7 (3-8) |

| **Lumbosacral radicular syndrome** | | **Delphi Round 1** | **Delphi round 1**  Median (range) | **Delphi Round 2**  Number of participants who rated a case definition ≥7 on a 9-point rating scale (N(%)). | **Delphi round 2**  Median (range) |
| --- | --- | --- | --- | --- | --- |
|  | Manual handling of heavy loads. | 61 (97%) | 7 (4-9) | 52 (93%) | 8 (4-9) |
|  | Bending or twisting of the trunk. | 58 (92%) | 7 (2-9) | 48 (86%) | 8 (5-9) |
|  | Whole-body vibration. | NA | NA | 42 (75%) | 7 (3-9) |
|  | Manual lifting. | NA | NA | 46 (82%) | 8 (4-9) |

| **Subacromial pain syndrome** | | **Delphi Round 1** | **Delphi round 1**  Median (range) | **Delphi Round 2**  Number of participants who rated a case definition ≥7 on a 9-point rating scale (N(%)). | **Delphi round 2**  Median (range) |
| --- | --- | --- | --- | --- | --- |
|  | Arm elevation (hand at or above shoulder height). | 62 (98%) | 8 (4-9) | 45 (80%) | 8 (5-9) |
|  | Repetitive work with hand and/or arm. | 61 (97%) | 7 (3-9) | 39 (70%) | 7 (5-9) |
|  | Daily work with vibrating hand tools. | 56 (89%) | 7 (2-9) | 38 (68%) | 7 (3-9) |
|  | Arm elevation (hand at or above shoulder height). | 57 (91%) | 7 (2-9) | 37 (66%) | 7 (1-9) |
|  | Combination of:  1. Repetitive work with hand and/or arm.  2. Daily work with vibrating hand tools.  3. High force of upper extremity. | NA | NA | 41 (73%) | 8 (3-9) |
|  | Manual tasks involving pushing or pulling loads. | NA | NA | 30 (54%) | 7 (3-9) |
|  | Arm elevation. | NA | NA | 31 (55%) | 7 (2-9) |
|  | Combination of:  1. Arm elevation (hand at or above shoulder height).  2. Repetitive work with hand and/or arm.  3. Daily work with vibrating hand tools.  4. High force of upper extremity. | NA | NA | 51 (91%) | 8 (4-9) |

| **Carpal tunnel syndrome** | | **Delphi Round 1** | **Delphi round 1**  Median (range) | **Delphi Round 2**  Number of participants who rated a case definition ≥7 on a 9-point rating scale (N(%)). | **Delphi round 2**  Median (range) |
| --- | --- | --- | --- | --- | --- |
|  | Repetition (frequency of exertion and duty cycle of exertion) of wrist/hand/fingers. | 61 (97%) | 8 (4-9) | 53 (95%) | 8 (5-9) |
|  | Force (peak effort exerted by the hand). | 61 (97%) | 7 (3-9) | 51 (91%) | 8 (4-9) |
|  | Combined exposures (repetition + force). | 60 (95%) | 8 (1-9) | 54 (97%) | 9 (6-9) |
|  | Vibration of the hand/arm. | 60 (95%) | 8 (4-9) | 49 (88%) | 7.5 (3-9) |
|  | Non-neutral wrist postures. | 56 (89%) | 7 (3-9) | 41 (73%) | 7 (3-9) |
|  | Working in cold environment. | 51 (81%) | 6 (2-9) | 30 (54%) | 7 (3-9) |
|  | External pressure on the carpal tunnel of the underarm. | NA | NA | 29 (52%) | 7 (3-9) |
|  | Fine manipulation with force. | NA | NA | 37 (66%) | 7.5 (4-9) |

| **Lateral elbow tendinopathy** | | **Delphi Round 1** | **Delphi round 1**  Median (range) | **Delphi Round 2**  Number of participants who rated a case definition ≥7 on a 9-point rating scale (N(%)). | **Delphi round 2**  Median (range) |
| --- | --- | --- | --- | --- | --- |
|  | Combination of turn and screw | 61 (97%) | 7 (3-9) | 48 (86%) | 8 (5-9) |
|  | Repetitive bending and twisting of the elbow | 60 (95%) | 7 (3-9) | 49 (88%) | 8 (3-9) |
|  | High physical exertion combined with elbow movements | 57 (91%) | 7 (3-9) | 45 (80%) | 8 (4-9) |
|  | Non-neutral elbow/under arm postures. | 55 (87%) | 7 (3-9) | 34 (61%) | 7 (3-9) |
|  | Use of vibrating hand tools. | 58 (92%) | 7 (3-9) | 39 (70%) | 7 (4-9) |
|  | Handling loads. | NA | NA | 29 (52%) | 7 (3-9) |
|  | High hand grip forces. | NA | NA | 36 (64%) | 7 (3-9) |
|  | Repetitive movements. | NA | NA | 33 (59%) | 7 (1-9) |
|  | Repetitive hand and wrist joint movements. | NA | NA | 40 (71%) | 7 (3-9) |
|  | High physical exertion of the wrist combined with elbow movements. | NA | NA | 42 (75%) | 7 (2-9) |
|  | A combination of all of the above:  A. Combination of turn and screw  B. Repetitive bending and twisting of the elbow  C. High physical exertion combined with elbow movements  D. Non-neutral elbow/under arm postures.  E. Use of vibrating hand tools. | NA | NA | 40 (71%) | 8 (3-9) |
|  | Twisting of the forearm/elbow. | NA | NA | 32 (57%) | 7 (2-9) |
|  | Forearm/elbow movements. | NA | NA | 23 (41%) | 5.5 (1-9) |

| **Medial elbow tendinopathy** | | **Delphi Round 1** | **Delphi round 1**  Median (range) | **Delphi Round 2**  Number of participants who rated a case definition ≥7 on a 9-point rating scale (N(%)). | **Delphi round 2**  Median (range) |
| --- | --- | --- | --- | --- | --- |
|  | Handling loads | 53 (84%) | 7 (2-9) | 38 (68%) | 7 (3-9) |
|  | High hand grip forces | 59 (94%) | 7 (4-9) | 45 (80%) | 8 (4-9) |
|  | Repetitive movements | 61 (97%) | 7 (4-9) | 42 (75%) | 7 (1-9) |
|  | Working with vibrating hand tools | 59 (94%) | 7 (3-9) | 40 (71%) | 7 (4-9) |
|  | Repetitive hand and wrist joint movements. | NA | NA | 37 (66%) | 7 (3-9) |
|  | High physical exertion of the wrist combined with elbow movements. | NA | NA | 40 (71%) | 7 (3-9) |
|  | A combination of:  1.Handling loads  2.High hand grip forces  3.Repetitive movements  4. Working with vibrating hand tools | NA | NA | 42 (75%) | 8 (3-9) |
|  | Exposure to abrupt mechanical stress (jolt). | NA | NA | 24 (43%) | 6 (3-9) |

| **Knee osteoarthritis** | | **Delphi Round 1** | **Delphi round 1**  Median (range) | **Delphi Round 2**  Number of participants who rated a case definition ≥7 on a 9-point rating scale (N(%)). | **Delphi round 2**  Median (range) |
| --- | --- | --- | --- | --- | --- |
|  | Lifting | 55 (87%) | 7 (1-9) | 40 (71%) | 7 (3-9) |
|  | Kneeling and/or squatting | 62 (98%) | 8 (3-9) | 52 (93%) | 8 (4-9) |
|  | Jumping | 60 (95%) | 7 (3-9) | 48 (86%) | 8 (4-9) |
|  | Climbing the stairs/ladder | 58 (92%) | 7 (1-9) | 48 (86%) | 8 (5-9) |
|  | Running. | NA | NA | 33 (59%) | 7 (2-9) |
|  | Heavy Lifting. | NA | NA | 44 (79%) | 7.5 (4-9) |
|  | Drop-jump. | NA | NA | 36 (64%) | 7 (3-9) |
|  | Standing on vertically moving standing platform (e.g., ship deck). | NA | NA | 26 (46%) | 6 (1-9) |

| **Hip osteoarthritis** | | **Delphi Round 1** | **Delphi round 1**  Median (range) | **Delphi Round 2**  Number of participants who rated a case definition ≥7 on a 9-point rating scale (N(%)). | **Delphi round 2**  Median (range) |
| --- | --- | --- | --- | --- | --- |
|  | Heavy lifting. | 58 (92%) | 7 (2-9) | 46 (82%) | 7.5 (1-9) |
|  | Work as a farmer. | 49 (78%) | 6 (1-9) | 34 (61%) | 7 (1-9) |
|  | Work as a construction worker. | 53 (84%) | 6 (1-9) | 35 (63%) | 7 (1-9) |
|  | Jockeys. | NA | NA | 21 (38%) | 6 (1-9) |
|  | Athletes. | NA | NA | 25 (45%) | 6 (1-9) |
|  | Miners. | NA | NA | 32 (57%) | 7 (1-9) |
|  | Drop-jump. | NA | NA | 29 (52%) | 7 (1-9) |
|  | Standing on vertically moving standing platform (e.g., ship deck). | NA | NA | 23 (41%) | 6 (1-9) |
|  | Standing. | NA | NA | 21 (38%) | 5 (1-9) |
